# HIV-1 envelope glycoprotein characteristics that correlate with the development of cross-reactive neutralizing activity in HIV-1 infected individuals

**DOI:** 10.1186/1742-4690-9-S2-P52

**Published:** 2012-09-13

**Authors:** TL van den Kerkhof, Z Euler, MJ van Gils, BD Boeser-Nunnink, NL Verwer, H Schuitemaker, RW Sanders

**Affiliations:** 1 Academic Medical Center University of Amsterdam, Amsterdam, the Netherlands

## Background

Cross-reactive neutralizing activity (CrNA) is elicited in around 30% of HIV-1 infected individuals and is most likely directed against conserved regions of the envelope glycoprotein complex (Env). We hypothesized that the induction of CrNA may at least to a certain extent depend on the phenotypic characteristics of Env from viruses early in infection.

## Methods

We selected 34 patients from the Amsterdam Cohort Studies (ACS) who had varying levels of CrNA at 2-4 years after seroconversion (SC). We retrospectively generated Env sequences from clonal HIV-1 variants that were isolated between 2 and 14 months after SC and analyzed the length of the variable regions and the number of potential N-linked glycosylation sites (PNGS).

## Results

For 31 out of 34 patients we observed a correlation between a higher level of CrNA and on one hand a shorter variable region 1 (P=0.04) and on the other hand a increased number of NXS sequons relative to the number of PNGS (P=0.04), which decreases the probability of glycosylation at that site. In contrast, in the 3 patients with the most potent CrNA, defined as elite neutralizers, the viral V1 region was longer with a higher number of NXT sequons relative to the number of PNGS. These viral characteristics are similar to those in patients with low potency of CrNA, rather than to those in patients with higher potency of CrNA in their serum.

## Conclusion

Our results suggest that in general the development of CrNA in HIV-1 infected patients is associated with a more open structure of the viral Env, mediated by a short V1 loop and a low probability of glycosylation. However, our three elite neutralizers were exceptions to this rule. The identification and understanding of Env characteristics that are involved in the development of CrNA should help the rational design of an effective antibody-based vaccine immunogen.

